# Disease Specific Aspects of Malnutrition in Neurogeriatric Patients

**DOI:** 10.3389/fnagi.2018.00080

**Published:** 2018-03-23

**Authors:** Tino Prell, Caroline Perner

**Affiliations:** Department of Neurology, Jena University Hospital, Jena, Germany

**Keywords:** malnutrition, geriatric, parkinson's disease, dementia, stroke

## Abstract

Malnutrition in elderly patients is a common condition. Nevertheless, there is evidence on specific risk factors and problems of malnutrition in geriatric patients with neurological diseases. In this review, we summarize recent knowledge on malnutrition in different neurological diseases with a focus on elderly patients. This overview also provides strategies for a more specific and profound assessment of neurogeriatric patients to improve identification and treatment of malnutrition. Early and consequent treatment of malnutrition can lead to a decreased progression of the neurological disease and to a better quality of life in geriatric patients.

## Introduction

As the burden of neurological disorders in the aging global population increases, there is an urgent need to pay more attention to geriatric syndromes in geriatric patients with underlying neurological disorders (neurogeriatric patients). An important and yet under-recognized geriatric syndrome is malnutrition. Although a widely accepted definition of malnutrition that adequately reflects its pathophysiology and its consequences is still lacking, the most relevant and recognized elements in the definition of malnutrition comprise the *deficiency of energy and of protein*, and the *decrease in fat-free mass* (Meijers et al., 2010). The European Society for Clinical Nutrition and Metabolism defines malnutrition as “a state resulting from lack of uptake or intake of nutrition causing altered body composition (decreased fat free mass and body cell mass), leading to diminished physical and mental function and impaired outcome from disease.” (Cederholm et al., 2015). Malnutrition has been shown to contribute to lower quality of life, an increase in the length of stay in hospital and increased rates of readmission, and higher morbidity and mortality (Rasheed and Woods, 2013; Tappenden et al., 2013). The prevalence of malnutrition in Europe and North America ranges from 1 to 22% with considerable variations between different settings (50.5% in rehabilitation; 38.7% in hospital; 13.8% in nursing home; 5.8% in community; Kaiser et al., 2010). However, the risk of malnutrition in older adults is expected to increase in parallel to the predicted global increase in life expectancy.

In clinical practice, the nutritional intake may be compromised in settings of: (1) pure chronic starvation without inflammation (e.g., anorexia nervosa), (2) conditions with sustained mild inflammation (e.g., cancer, rheumatoid arthritis), and (3) acute disease with significant inflammation which elevates resting energy expenditure (e.g., pneumonia) (Jensen et al., 2010). The catabolic effect of the inflammatory response in critical illness promotes makes it more difficult for geriatric patients to compensate hypocaloric episodes (Jensen et al., 2010). It is therefore important to notice that inflammatory conditions can be chronic in nature of some neurodegenerative disorders, aggravating loss of muscle mass and function over many months or even years. The condition when inflammation leads to a decrease in lean body mass that is associated with functional impairment is called “disease-related malnutrition” (DRM). Malnutrition can therefore occur as DRM or starvation (food deprivation). DRM can be further categorized as cachexia (inflammatory-induced DRM in the presence of acute or chronic diseases) or as non-cachectic DRM (without inflammation) (White et al., 2012; Malone and Hamilton, 2013). Since many neurological and neurodegenerative disorders are accompanied by inflammatory processes, these diseases could inherently lead to malnutrition.

The aim of this narrative review is to summarize current knowledge on the physiological, pathological, and social mechanisms of malnutrition and to discuss the disease-specific aspects of malnutrition in neurogeriatric patients.

## Assessment and screening for malnutrition

A gold standard for the optimal definition of malnutrition is still lacking. However, an ideal nutritional marker would need to have high specificity and sensitivity), not affected by non-nutritional factors, and normalize through nutritional therapy (Harris and Haboubi, 2005). A body mass index (BMI) (weight[kg]/height[m^2^]) <20–22 is usually regarded as indicator for malnutrition in geriatric patients (Volkert et al., 2006). However, BMI is influenced by hydration and may be unreliable in the presence of oedema. Since malnutrition also occurs in patients with a normal BMI, the BMI alone is not an appropriate/adequate parameter to predict malnutrition. Nevertheless, changes in BMI and weight loss offer the dynamic dimension and include aspects of anorexia, dental problems, dysphagia, and insufficient food intake. Moreover BMI is useful as a longitudinal marker for monitoring nutritional therapy. On the other hand, anthropometric indices are simple to obtain but can be unreliable in limb oedema. The *arm circumference* is used because it depends on the mass of the muscle group is proportional to its protein content and reflects total body muscle mass (Harris and Haboubi, 2005). *Mid-upper arm circumference below* 23 cm (males)/22 cm (females) *and calf-circumference below 31 cm are sensitive* indicators of malnutrition and easily applicable (Bonnefoy et al., 2002). *Skinfold thickness* which can be measured with standardized calipers can be used for different body regions. The skinfold thickness of triceps is widely used and represents a long-term marker for body fat (Corish and Kennedy, 2003). In addition, several serum proteins, such as albumin, prealbumin, and transferrin have been used as biochemical markers of nutrition. An albumin level below 35 g/dl is widely regarded as a threshold marker of malnutrition in older people. However, its usefulness is limited in inflammatory conditions and in liver and kidney diseases. Moreover, it does not reflect short-term changes in protein and energy intake (Jeejeebhoy et al., 1982; Kuzuya et al., 2007). Further, since malnutrition also contributes to immune dysregulation, a decreased *total lymphocyte count* frequently occurs in malnutrition (Gariballa and Sinclair, 1998; Gariballa, 2004).

The European Society for Clinical Nutrition and Metabolism (ESPEN) recommends three scores for screening malnutrition. The Malnutrition Universal Screening Tool (MUST) uses BMI, history of unexplained weight loss and acute illness effect (Malnutrition Advisory Group, 2000; Elia, 2003). MUST was originally developed for use in the community (e.g., for predicting admission rates) but it has recently been extended for use in other health care settings including hospitals. Having excellent inter-rater reliability, and concurrent validity with other tools it is also predictive in terms of length of hospital stay and mortality in elderly (Kondrup et al., 2003a). The Nutritional Risk Screening (NRS-2002) was developed to detect the presence of malnutrition in adults in the hospital setting. It contains components of MUST and a grading scale for severity of disease (Kondrup et al., 2003b). The Mini Nutritional Assessment (MNA) evaluates the presence of malnutrition and the risk of developing malnutrition among the elderly in home-care programs, nursing, homes and hospitals (Bauer et al., 2008; Kaiser et al., 2009). Since it includes physical and mental aspects that frequently affect the nutritional status it is more reliable for identifying the risk of developing malnutrition (Kondrup et al., 2003a). Moreover, the NMA detects the risk of malnutrition when albumin levels and BMI are still normal. The score is derived from six items—reduced food intake in the preceding 3 months; weight loss during the preceding 3 months; mobility; psychological stress or acute disease in the preceding 3 months; neuropsychological problems; and body mass index. The MNA is highly predictive for adverse health outcome, social functioning, length of hospital stay and mortality (Harris and Haboubi, 2005).

## General reasons for malnutrition

Similar to other geriatric syndromes, malnutrition is also a syndrome with multifactorial genesis. In most cases, malnutrition is based on insufficient food intake and less frequently on a higher need of nutrients or on a problem of malassimilation. During aging a series of physiological changes take place which favor malnutrition. These changes are usually summarized under the term *anorexia of aging*, defined as the loss of appetite and/or decreased food intake in late life (reviewed in Landi et al., 2016). As a result, the compensation of hypocaloric episodes, e.g., during acute illness is rendered more difficult in geriatric patients. A recent systematic review of 6 longitudinal studies reported the following significant risk factors for malnutrition: age, frailty in institutionalized persons, excessive polypharmacy, general health decline including physical function, Parkinson's disease, constipation, poor or moderate self-reported health status, cognitive decline, dementia, eating dependencies, loss of interest in life, poor appetite, basal oral dysphagia, signs of impaired efficacy of swallowing, and institutionalization (Fávaro-Moreira et al., 2016). Impaired motor skills and sensory and cognitive deficits such as stroke, Parkinson's disease or dementia are frequently observed in neurogeriatric patients. These conditions, often accompanied by poorer oral health and dental hygiene, increase the risk of malnutrition and—if there is also dysphagia—pneumonia (Müller et al., 2011). Moreover, the chronic inflammatory conditions in periodontal disease have been linked to progression of neurodegenerative disorders, such as Alzheimer's or Parkinson's disease (Kaur et al., 2016). Common factors which contribute to malnutrition are listed in Table 1.

**Table 1:** General factors contributing to anorexia of aging and malnutrition.

**GASTROINTESTINAL CHANGES**
decreased appetite and thirst decreased sense of smell and taste (diminished food intake) decline in saliva secretion (reduce the ability to dissolve foods) poor dentition abnormalities in gastric motility (loss of gastric compliance and more rapid antral filling, delayed gastric emptying; proton-pomp inhibitors can further delay gastric emptying) constipation (due to immobility, chronic volume deficiency, low-fiber diet, and inadequate hydration)
**SYSTEMIC CHANGES**
hormonal changes (g*hrelin, cholecystokinin, peptide YY, leptin*) presence of chronic low-grade inflammation (increased circulating levels of interleukin 1, 6, and tumor necrosis factor alpha) leading to reduced food intake reduced energy requirements (reduced physical activity, reduced food intake)
**GERIATRIC SYNDROMES**
multimorbidity: acute diseases (can lead to temporary anorexia resulting in loss of weight, which often cannot be compensated), chronic infections (congestive heart failure, chronic obstructive pulmonary disease, Parkinson's disease), tumor cachexia, pulmonary cachexia, renal cachexia) depression and dementia (loss of appetite and reduced food intake) drug-induced anorexia polypharmacy social isolation functional impairments in activities of daily living (ADL) (reduced food intake and loss of appetite) sensory and motor impairment (e.g., problems in eating by oneself, difficulty in getting foods, and lack of cooking skills)

## Disease-specific aspects of malnutrition in neurogeriatric patients

Besides the general risk factors for malnutrition mentioned above, there is evidence for disease specific aspects and predictors for malnutrition in neurogeriatric patients (summarized in Table 2). To know which patient is at high risk of malnutrition is highly relevant in daily routine in order to perform an effective screening and to start interventions at the right time.

**Table 2:** Disease specific risk factors and predictors of malnutrition.

**Parkinson's disease**	greater age baseline weight female gender higher baseline Unified Parkinson Disease Rating Scale (UPDRS) greater postural instability lower cognitive scores baseline levodopa use difficulty in eating and drinking
**Stroke**	dysphagia Immobility complications (e.g., pneumonia, thrombosis, depression)
**ALS**	depression excessive salivation dyspnea dysphagia difficulty with self-feeding and preparing meals due to paresis constipation cognitive decline
**Epilepsy**	antiepileptic drugs (felbamate, topiramate, zonisamide)
**Multiple sclerosis**	dysphagia

### Parkinson's disease

Weight loss is a frequent non-motor syndrome in patients with Parkinson's disease and is associated with impaired quality of life. Weight loss can also precede the motor stage. Whilst the prevalence of weight loss ranges from 0 to 24%, between 3 and 60% of patients with Parkinson's disease are reported to be at risk of malnutrition (Sheard et al., 2011; Tomic et al., 2017). In particular reduced energy intake due to dysphagia, poor dental status, gastrointestinal dysfunction and increased energy expenditure due to rigidity, tremor, and dyskinesia but also dementia, anxiety and depression have been found to be associated with malnutrition in Parkinson's disease (Beyer et al., 1995; Kashihara, 2006; Lorefält et al., 2009; Sheard et al., 2013; Fávaro-Moreira et al., 2016; Lindskov et al., 2016; Mukherjee et al., 2016).

In a recent analysis of longitudinal data (1–6 years) from 1619 participants of greater age, baseline weight, female gender, higher baseline disease severity assessed by Unified Parkinson Disease Rating Scale (UPDRS) scores, greater postural instability, difficulty in eating and drinking, lower cognitive scores, and baseline levodopa use (compared to dopamine agonists) were all associated with weight loss. Surprisingly difficulties such as swallowing, dyskinesia, depression, constipation, and nausea/vomiting were not significantly associated with weight loss in this cohort of early treated Parkinson's disease patients (Wills et al., 2017). In fact, in this large cohort, people with early stage Parkinson's disease experienced very little weight loss (on average about 0.6 kg/year) during the first 1–6 years after starting dopaminergic replacement therapy. However, while the majority of participants did not experience significant weight loss, a minority (7.8%) did lose at least 5% of their body weight over the first year of the study. With reference to a former study by Will et al. participants who experience a decline in BMI also experience a more rapid worsening of motor function (UPDRS III) (Wills et al., 2016). Whether therapy with dopamine agonists additionally influences nutritional status through adverse events, such as nausea, vomiting or anorexia remains controversial. There are however no robust data concerning the question of how different agonists impact nutritional status. Some anorexia nervosa studies have reported that dopamine D1 and D2 receptor stimulation could be a helpful pharmacological intervention for improving the re-feeding process (Tomic et al., 2017). On the other hand, stimulation of D3 receptors seems to play a major role in impulsive behavior disorders including impulsive eating. In this context, one could presume that agonists acting on D2 receptors influence nutrition more efficiently (Tomic et al., 2017).

Along with low body weight, PD patients often have a lower bone mineral density than age-matched controls. Vitamin D deficiency is also common in Parkinson's disease and reduces bone mineral density (van den Bos et al., 2013; Lv et al., 2014). Lastly, due to levodopa interaction with the methylmalone pathway, hyperhomocysteinaemia, an independent risk factor for osteoporosis, is common in Parkinson's disease, as well as in vitamin B12 and folic acid deficiency (van den Bos et al., 2013).

### Stroke

Due to the high prevalence of dysphagia, immobility and complications (e.g., pneumonia, thrombosis, depression) patients with acute stroke are at a high risk for developing malnutrition. The National Institute for Health and Care Excellence (NICE) guideline states the necessity of screening and assessing all acute stroke patients for swallowing problems by appropriately trained staff before being given any oral food, fluid or medication (Ojo and Brooke, 2016). In acute stroke, the prevalence of dysphagia has been reported as ranging from 28 to 65%, depending on the method used for assessing dysphagia. In addition, dysphagia is reported to improve significantly during the early days following stroke and after 2 weeks 90% of patients have regained their ability to swallow (Cohen et al., 2016). Nevertheless complications associated with dysphagia include aspiration, pneumonia, recurrent cough, choking, increased length of hospital stay, increased rehabilitation time and increased mortality (Sura et al., 2012) Although post-stroke dysphagia increases the odds of malnutrition (Foley et al., 2009), factors e.g., level of physical ability and cognitive decline that might have been present before the occurrence of stroke should be taken into account when assessing nutritional status. For example, approximately 16% of stroke patients present with nutritional deficits upon admission. During acute hospitalization and after discharge from hospital, nutritional deficits may worsen and therefore malnutrition is more prevalent after the acute stroke phase, with a reported prevalence of up to 45% during the rehabilitation phase (Axelsson et al., 1988; Dávalos et al., 1996; Gariballa et al., 1998; Sura et al., 2012).

### Epilepsy

Beside the well-known factors for malnutrition mentioned above, specific neurological drug-induced anorexia is an important issue to consider when talking about malnutrition in neurogeriatric patients. The administration of antiepileptic drugs (AEDs) is higher in the elderly than in other adults. This is caused by an increased prevalence of epilepsy, neuropathic pain and behavioral disorders associated with dementia, for which AEDs are frequently administered. Extensive use of AEDs accounts for adverse drug reactions and contributes to the high frequency of untoward drug effects (Lackner, 2002). Since, treatment with AEDs is often long-term over many years, physicians should be aware of the metabolic changes associated with AED use. AEDs can be linked to weight gain (gabapentin, pregabalin, valproate, vigabatrin) which increases risk for obesity-related vascular disorders or weight loss (felbamate, topiramate, zonisamide) and risk for malnutrition (Ben-Menachem, 2007). Felbamate causes significant weight loss in long term follow up and has considerable toxicity and therefore it's application is usually restricted to the Lennox-Gastaut syndrome (Bergen et al., 1995; Cilio et al., 2001). Topiramate is indicated for epilepsy and migraine prevention. Several studies reported that this AED is associated with weight loss (Freitag et al., 2002; Ben-Menachem et al., 2003; Wilding, 2004; Arroyo et al., 2005; Stenlöf et al., 2007). Weight loss seems to be most evident in patients with BMI >30 and is the result of losses in body fat and decreased food intake (Ben-Menachem et al., 2003). Zonisamide is well tolerated broad-spectrum AED. Weight loss is consistently reported in patients receiving Zonisamid and ranges between 2 and 9.2 kg (Faught et al., 2001; Gadde et al., 2003). Alterations of bone metabolism leading to decreased bone mineral density can worsen the risk for fractures, via seizure-related falls and trauma (Sheth, 2004). Continuous use of AEDs in elderly women is associated with increased rates of bone loss at the calcaneus and hip (Ensrud et al., 2004). The CYP450 enzyme-inducing AEDs such as phenytoin, phenobarbital, carbamazepine, and primidone are frequently associated with bone disorders. The data regarding the effect of valproate and newer AEDs such as lamotrigine, gabapentin, vigabatrin, levetiracetam, and topiramate on bone metabolism and bone density remain controversial (Arora et al., 2016). Currently, there are no valid data addressing the issue of malnutrition in geriatric patients with epilepsy. Further research needs to be conducted to study the epidemiology and disease specific predictors of malnutrition in these patients.

### Multiple sclerosis

Nutritional problems have also been found to be associated with multiple sclerosis (MS). Although it is reasonable to expect that due to the nature of MS (functional deficits, cognitive and psychological disturbances, neuroinflammatory processes) malnutrition is associated with MS, the prevalence of malnutrition in elderly MS patients is currently unknown (Kamalian et al., 1975; Fantelli et al., 1978; Williams et al., 1988; Thomas and Wiles, 1999). A retrospective study did not show a significant difference in the frequency of malnutrition (here only defined as albumin below 3.5 g/dl) in MS patients when compared with other chronic pathologies. The lower albumin was independent of disease course, disease duration, number of attacks, disability status and functional system involvement (Sorgun et al., 2014). Nortvedt et al. reported that patients with MS seem to have a lower mean BMI (which alone is not a good indicator for malnutrition) than the recommended range despite low leisure physical activity (Nortvedt et al., 2005). Similarly, Alschuler et al. reported lower BMI in patients with MS in relation to the general population (Alschuler et al., 2012). Lower BMI is more often observed in female MS patients (Khurana et al., 2009; Markianos et al., 2013). However, all the above data need to be confirmed with further prospective studies in larger MS populations from differing facilities. Moreover, existing studies did not focus on elderly MS patients and taking all studies under consideration, there are not yet enough data to evaluate the risk/presence of malnutrition in geriatric MS patients. Similar to Parkinson's disease, several studies indicate that patients with MS have a high incidence of osteopenia and osteoporosis and vitamin D deficiency with consequently higher risk of falls and immobility (Bagur et al., 2017).

Dysphagia is a frequently underestimated symptom in patients with MS. Depending on the method used for assessing the swallowing function, the prevalence of dysphagia ranges between 10 and 90% (Fernandes et al., 2013). A systematic review and meta-analysis defining the aggregate prevalence of dysphagia in multiple sclerosis in a total of 4,510 subjects from fifteen eligible studies revealed that overall prevalence rates of 36% for subjective assessment and 81% for objective assessment, suggesting that at least one-third of the MS patients were suffering from dysphagia (Guan et al., 2015). Dysphagia reduces the quality of life and increases the risk of dehydration and aspiration pneumonia. These disturbances mainly involve oral and pharyngeal phases of swallowing. Abnormal swallowing was associated with several factors including abnormal brainstem/cerebellar function, higher EDSS scores, vital capacity, depression score, progressive forms of MS, and long disease duration (Thomas and Wiles, 1999; Poorjavad et al., 2010; Guan et al., 2015). The high prevalence of dysphagia emphasizes the importance of the assessment of the swallowing function in MS patients.

### Amyotrophic lateral sclerosis

Amyotrophic lateral sclerosis is a neurodegenerative, multisystemic disorder characterized by progressive loss of motor function due to motoneuronal dysfunction. Additionally cognitive disturbances with an overlap to frontotemporal dementia are frequently observed. The mean survival of ALS patients is 3–5 years and death is usually due to respiratory failure. Malnutrition is common in ALS patients and it negatively affects prognosis, survival and quality of life (Limousin et al., 2010; Marin et al., 2011). Each 5% unit decrease in usual BMI was found to be correlated with an adjusted 24% increased risk of death (Marin et al., 2011). To prevent malnutrition in ALS it is therefore necessary to positively influence survival and quality of life (Park and Kang, 2009). The European ALS Consortium and the American Academy of Neurology recommend a proactive nutrition management and a regular nutrition assessment every 3 months (Andersen et al., 2007; Miller et al., 2009). Disease-specific risk factors for malnutrition in ALS are depression, excessive salivation, dyspnea, difficulty with self-feeding and obtaining/preparing meals due to paresis, constipation, cognitive decline, and dysphagia (Greenwood, 2013). To maintain oral nutritional intake in case of swallowing dysfunction, food consistency should be adapted (blending food, adding thickeners to liquids). Moreover, patients and caregivers should be educated in feeding and swallowing techniques (EFNS Task Force on Diagnosis and Management of Amyotrophic Lateral Sclerosis et al., 2012). In general, altered food consistency is usually associated with lower calories and therefore additional high-protein and high-caloric supplements should be considered. Ultimately, a percutaneous endoscopic gastrostomy (PEG) or percutaneous radiologic gastrostomy (PRG, or radiologically inserted gastrostomy) may be needed as an alternative route for delivering nutrients. It is important to emphasize to patients that PEG still allows oral feeding but offers the possibility to administer medication, fluids and nutrition (Miller et al., 2009). PEG improves nutrition, but there is no convincing evidence as yet that it prevents aspiration or improves quality of life (EFNS Task Force on Diagnosis and Management of Amyotrophic Lateral Sclerosis et al., 2012). The introduction and timing of PEG is based on an individual approach and should take into consideration that the risk of respiratory complications increases in patients with severe ventilatory muscle impairment (Sancho et al., 2010). To minimize risks, PEG should therefore be performed before vital capacity falls below 50% of predicted value (EFNS Task Force on Diagnosis and Management of Amyotrophic Lateral Sclerosis et al., 2012). Further widely accepted indications for the placement a feeding tube include: (a) weight loss of 5–10%, (b) BMI < 20 kg/m2, and (c) inability to take in adequate nutrition and hydration (Shaw et al., 2006; Greenwood, 2013). A large prospective study of 345 patients with ALS showed that overall mortality was independent of gastrostomy method but driven by disease progression and the level of care at treatment centers (McDermott et al., 2015).

### Dementia

Dementia is a neurological symptom characterized by a long term decrease in cognitive abilities such as the ability to think and remember that affects the daily functioning of patients. The most common types of dementia are Alzheimer's disease (50–70%), vascular dementia (25%), Lewy body dementia (15%), and frontotemporal dementia (Burns and Iliffe, 2009).

There is much evidence that dementia is a risk factor for malnutrition (Meijers et al., 2014). In geriatric patients, the prevalence of malnutrition is 10% higher in patients who suffer from dementia (Reuther et al., 2013). The reasons for this are complex. Patients with dementia often have higher care dependency rates and require more assistance for eating and drinking which in turn also leads to a higher rate of patients who are institutionalized and dependent on help. This dependency may affect patient's emotional and psychological well-being. The nutritional status and nutritional care of patients with dementia has also been reported as being associated with their psychological well-being (Muurinen et al., 2015). Moreover, some reports describe increased malnutrition in patients with dementia compared to elderly patients without because demented patients seem to have higher nutritional requirements (Galesi et al., 2013). An adequate diagnosis of malnutrition and the identification of modifiable factors such as functional impairment, eating behaviors and emotional well-being/depression are important to implement a specific treatment to avoid malnutrition (Roqué et al., 2013).

## Therapy of malnutrition

The prevention and treatment of malnutrition depends on its etiology and may be accomplished through multiple interventions, such as correction of environmental and pharmacological risk factors, treatment of underlying medical causes or via energy supplementation. It is obvious that all medical causes (such as tooth loss or dysphagia) have to be specifically addressed. The therapeutic goals in geriatric patients do not fundamentally differ from those for younger patients, but are weighted differently. While in younger patients the reduction of morbidity and mortality is of importance, the preservation of function, independence and quality of life are the focus in geriatric patients (Volkert et al., 2013). Therefore, nutritional therapy in geriatric patients includes a wide range of different apporaches. Oral strategies have a top priority for older people. However, if these oral strategies fail to provide adequate food intake, oral nutritional supplements and probe nutrition provide the opportunity to secure or increase energy and nutrient intake. In general, parenteral nutrition should be reserved for patients who are not able to meet their oral needs via the enteral route (Volkert et al., 2013). In terms of timing enteral feeding in stroke patients, it seems that early initiation (i.e., in <7 days) of enteral feeding was associated with better survival compared with late initiation (ie, in >7 days). PEG and nasogastric tube feeding do not differ in terms of composite outcome of death or dependency, but PEG is associated with fewer complications, and higher feed delivery and albumin concentration (Geeganage et al., 2012).

Since there are several national and international guidelines and reviews dealing with nutritional therapy in neurological as well as geriatric patients, detailed recommendations are not included here (e.g., Volkert et al., 2006, 2013; Sobotka et al., 2009; EFNS Task Force on Diagnosis and Management of Amyotrophic Lateral Sclerosis et al., 2012; Stavroulakis and McDermott, 2016). While there are recommendations for nutritional (enteral or parenteral) support for stroke and ALS patients, there is no evidence on the effectiveness of enteral feeding on the improvement of the nutritional status, quality of life or survival of patients with Multiple Sclerosis or Parkinson's disease (Stavroulakis and McDermott, 2016).

For the Neurologist it is important to evaluate pharmacological therapies in order to identify drugs that may favor malnutrition: (1) felbamate, topiramate, zonisamide; (2) cardiovascular drugs (digoxin, amiodarone, spironolactone); (3) psychiatric drugs (lithium, amitriptyline, selective serotonin reuptake inhibitors), and (4) non-steroidal anti-inflammatory agents (Landi et al., 2016). It is also of importance to notice that oropharyngeal dysphagia in neurogeriatric patients is a highly prevalent and serious problem and still underdiagnosed and untreated (Baijens et al., 2016). Therefore, the Neurologist should keep in mind the known disease specific risk factors and predictors of dysphagia and malnutrition in order to develop proactive nutrition management in neurogeriatric patients.

## Author contributions

All authors listed have made a substantial, direct and intellectual contribution to the work, and approved it for publication.

### Conflict of interest statement

The authors declare that the research was conducted in the absence of any commercial or financial relationships that could be construed as a potential conflict of interest.
